# Experimental research on the influence of acid on the chemical and pore structure evolution characteristics of Wenjiaba tectonic coal

**DOI:** 10.1371/journal.pone.0301923

**Published:** 2024-04-23

**Authors:** Xianxian Li, Xijian Li, Enyu Xu, Honggao Xie, Hao Sui, Junjie Cai, Yuhuan He

**Affiliations:** 1 College of Mining, Guizhou University, Guiyang, China; 2 Guizhou Engineering Center for Safe Mining Technology, Guiyang China; 3 College of Resource and Environmental Engineering, Guizhou University, Guiyang, China; Universiti Teknologi Petronas: Universiti Teknologi PETRONAS, MALAYSIA

## Abstract

The chemical and pore structures of coal play a crucial role in determining the content of free gas in coal reservoirs. This study focuses on investigating the impact of acidification transformation on the micro-physical and chemical structure characteristics of coal samples collected from Wenjiaba No. 1 Mine in Guizhou. The research involves a semi-quantitative analysis of the chemical structure parameters and crystal structure of coal samples before and after acidification using Fourier Transform infrared spectroscopy (FTIR) and X-ray diffraction (XRD) experiments. Additionally, the evolution characteristics of the pore structure are characterized through high-pressure mercury injection (HP-MIP), low-temperature nitrogen adsorption (LT-N_2_A), and scanning electron microscopy (SEM). The experimental findings reveal that the acid solution modifies the structural features of coal samples, weakening certain vibrational structures and altering the chemical composition. Specifically, the asymmetric vibration structure of aliphatic CH_2_, the asymmetric vibration of aliphatic CH_3_, and the symmetric vibration of CH_2_ are affected. This leads to a decrease in the contents of -OH and -NH functional groups while increasing aromatic structures. The crystal structure of coal samples primarily dissolves transversely after acidification, affecting intergranular spacing and average height. Acid treatment corrodes mineral particles within coal sample cracks, augmenting porosity, average pore diameter, and the ratio of macro-pores to transitional pores. Moreover, acidification increases fracture width and texture, enhancing the connectivity of the fracture structure in coal samples. These findings provide theoretical insights for optimizing coalbed methane (CBM) extraction and gas control strategies.

## 1 Introduction

China possesses the third-largest recoverable coal resources and the highest coal production globally. However, the country faces serious challenges, particularly in Guizhou, where coal and gas outburst accidents are frequent [1, 2]. Studies show that CBM extraction and utilization present an opportunity to address natural gas resource shortages and significantly reduce the risk of gas outbursts [3]. Tectonic deformation influences the original physical structure and chemical properties of coal, making tectonic coal a crucial indicator for determining the gas prominence of coal seams [4]. It is worth noting that tectonic coal contains numerous micropores and cracks, significantly impacting CBM adsorption and desorption. More specifically, tectonic coal is especially rich in medium pores (100–1000 nm) and large pores (>1000 nm) [5, 6]. Its large porosity and good connectivity make it favorable for the CBM development [7, 8]. China holds substantial CBM reserves, with 30.05×10^12^ m^3^ of CBM geological resources with an average gas content of 5.66 m^3^/t at burial depths less than 2000m [9]. Guizhou Province ranks second in the country with CBM geological resources of 3.06×10^12^ m^3^. The annual CBM production in Guizhou Province increases by 0.3213×10^8^ m^3^ and is expected to reach 0.9×10^8^ m^3^ in 2022 [10, 11]. Accordingly, understanding the pore characteristics of coal is crucial for comprehending the behavior of coal regarding CBM adsorption, desorption, and diffusion. The study of microphysical-chemical structural characteristics of tectonic coals is of significant importance in understanding CBM desorption [12–14].

Acid modification is a crucial chemical method for enhancing oilfield gas extraction and is particularly significant for corroding mineral-filled tectonic coal pores and fractures, thereby improving their connectivity [15, 16]. Studies show that acidification pretreatment of coal yields several benefits, including improvements in equilibrium adsorption and enhanced Nuclear Magnetic Resonance Spectroscopy (NMR) signals in the saturated water state [17, 18]. For instance, pretreatment of low-rank coal with acetic and hydrofluoric acid increases its hydrophobicity [19]. Hydrochloric acid has been widely used to remove minerals from lignite, increase the number of side chains, and alter pore structure and aromaticity [20, 21]. Designing an appropriate acidification system is of significant importance in modifying the pore and fracture structure of tectonic coals [22, 23]. After acidification treatment, the physicochemical structure of tectonic coals changes. The microscopic physicochemical structure of coal is generally categorized into pore structure (physical) and molecular structure (chemical) [24, 25]. Current quantitative analyses of the pore structure of tectonic coals primarily involve methods such as high-pressure mercury intrusion (HP-MIP), LT-N_2_A, low-temperature carbon dioxide adsorption (LTCO_2_-GA), and nuclear magnetic resonance (NMR). These methods provide valuable information on pore volume, specific surface area, the most available number of pore diameters, and pore size distributions, enabling comprehensive and accurate characterization of the tectonic coal structure [26–29]. The challenge in studying the pore and fracture structure of tectonic coal lies in the diverse principles and outcomes of different experimental methods. Porosity size, determined by various techniques, may yield different results, necessitating a comprehensive approach that combines multiple experiments to analyze the complex structure of tectonic coal. Observing the connectivity and filling state of the pore and fracture structure typically involves direct visualization using transmission electron microscopy, atomic force microscopy, and SEM. While these techniques provide valuable insights into the geometrical and morphological representation of pore structure within tectonic coal, quantitative analysis remains challenging [30–33]. Furthermore, the molecular structure (chemistry) of the pore and cleavage structure of tectonic coal is explored through various experiments, including Fourier infrared spectroscopy, XRD, Raman spectroscopy, and photoelectron spectroscopy (XPS). These methods contribute to a comprehensive understanding of the chemical structure of tectonic coal, focusing on functional groups and mineral composition [34–37]. Historically, acidification treatments have been applied predominantly to low-rank coals, with limited studies on medium and high-rank tectonic coals.

Presently, acid treatment is employed to enhance pore connectivity in coal samples from low-permeability coal seams in Guizhou. Many studies have only focused on the use of a singular type of acid to investigate alterations in pore or chemical structure, while limited investigations have been conducted on the application of mixed acids for this purpose [38, 39]. To explore the chemical and pore structure evolution of Wenjiaba tectonic coal, this study employs a mixed acid solution consisting of 15% hydrochloric acid (HCl) and 5% hydrofluoric acid (HF) for coal sample acidification. The analysis involves the use of FTIR and XRD to examine the chemical structure of tectonic coals before and after acidification, with a quantitative assessment of functional group characteristics, chemical structure parameters, and coal sample crystalline structure. The pore and fracture structure of the coal pre- and post-acidification is characterized using HP-MIP and LT-N_2_A. Additionally, changes in pore structure are visually observed using SEM, adhering to ISO/standard for physical and chemical analyses of pore structure. The study aims to analyze the evolution of the physical and chemical structure of tectonic coal under acidification, providing theoretical insights to enhance CBM extraction and gas control practices.

## 2 Coal samples and experimental methods

### 2.1 Collection and preparation of coal samples

#### 2.1.1 Coal sampling

The coal specimens were collected from the No. 7 coal seam situated in the 110705 working face of the Wenjiaba Coal Mine, located in Zhijin County, Bijie City, Guizhou Province (Longitude: 105.76309, Latitude: 26.66049398).

Following geological tectonic actions, such as extrusion, shearing, and crushing, the initial coal seam undergoes various stresses, leading to the disruption of its original stratification and structure. This results in the pulverization of the primary structure. The pulverized primary structural coal subsequently experiences additional tectonic stresses, facilitating the formation of tectonic coal with low strength and weak adhesion. When subjected to mining activities, loose tectonic coal particles are produced, exhibiting characteristics such as low strength, easy fracturing, and a lackluster appearance. Primary coal refers to coal that retains its original primary sedimentary structure and tectonic features even after coalization and metamorphic processes. On the other hand, tectonic coal experiences changes in composition, structure, and tectonics due to tectonic stress. In this study, a comparative analysis is conducted on the macrostructure of primary coal and tectonic coal originating from the same mining area, and the results are presented in Table 1.

**Table 1:** Macrostructure of primary and tectonic coal.

Coal samples	Macrostructures
Primary coal	Zonal jet, Linear structure, Lentoid, Homogeneous structure, leaf-like texture
Tectonic coal	Fragmentation structure, Grain structure, Silt structure, Mylonitic structure

Table 1 indicates that the structure of primary coal in the study area is relatively robust and intact compared to tectonic coal. Most of the tectonic coals exhibit a fractured and powdery granularity. Acidification experiments were conducted on the selected tectonic coals in the study area to investigate the impact of acidification on the development of the pore and fracture structure of tectonic coals.

#### 2.1.2 Parameters of the coal sample

Proximate analysis and ultimate analysis were conducted on the coal samples. To this end, an elemental analyzer (Elementar vario EL, Germany) was utilized and the test results are presented in Table 2.

**Table 2:** Measured parameters of coal samples.

Coal samples	Proximate analysis (%)				Ultimate analysis (%)			
	M_ad_	A_ad_	V_ad_	F_Cad_	C	N	H	S
Wenjiaba tectonic coal	2.83	25.06	8.83	63.28	70.3025	0.9885	1.9335	2.324

Based on the coal classification standard [40], Wenjiaba tectonic coal is categorized as semi-anthracite coal. In the elemental analysis table, apart from carbon (C), the content of other elements is below 10%, indicating a relatively stable composition for each element.

#### 2.1.3 Preparation of coal samples

The lumpy coal samples were initially crushed, followed by grinding using an onyx mortar and pestle. Subsequently, an XSB-88 vibrating sieve machine was employed to screen particles within the range of 0.25 to 0.45 mm (Particle sieving shall be conducted in accordance with GB/T 477). Concurrently, molds were utilized to create standard coal samples measuring 1 mm × 1 mm × 1 mm [41, 42]. The resulting samples were then placed in a drying box at 60°C.

### 2.2 Experimentation and methodology

#### 2.2.1 Experimental program

The coal samples were prepared through the following procedures: (1) Grinding the coal samples collected from the study area and preparing standard samples measuring 1 mm × 1 mm × 1 mm using molds; (2) Formulating a suitable acid mixture based on research findings [43, 44] to achieve optimal acidification; (3) Conducting acidification experiments on the coal samples; (4) Employing a FT-IR for the analysis of the microscopic molecular structure and macroscopic chemical composition of tectonic coal; conducting XRD experiments to analyze the crystal structure of coal samples before and after acidification. The infrared spectroscopy experiment parameters were set as follows: resolution 4 cm^-1^, number of scans 16, scanning range 4000~400 cm^-1^. Moreover, the XRD parameters were as follows: tube pressure 40 KV, diffraction width DS = SS = 1°, scanning speed 2.000 (d/min), scanning range 10°~80°; (5) Utilizing HP-MIP and LT-N_2_A experiments to analyze the pore structure of tectonic coal. The high-pressure mercury pressure test had a pressure range of 0.03–2200 MPa, and the pore size determination range was 350–0.005 μm. Cryogenic liquid nitrogen experiment parameters were set as follows: liquid nitrogen concentration 0.808 g/cc, experiment temperature: 77.350 K; (6) Using SEM to examine the changes in the pore space of coal samples before and after acidification. Adjustments to contrast and brightness were made during SEM experiments, with contrast set at about 60, brightness at about 0–20, and the bias beam current of the bulb adjusted to approximately 100 uA. The following section analyzes the experiments and their results.

#### 2.2.2 Proportioning of acid

**Fig 1:**
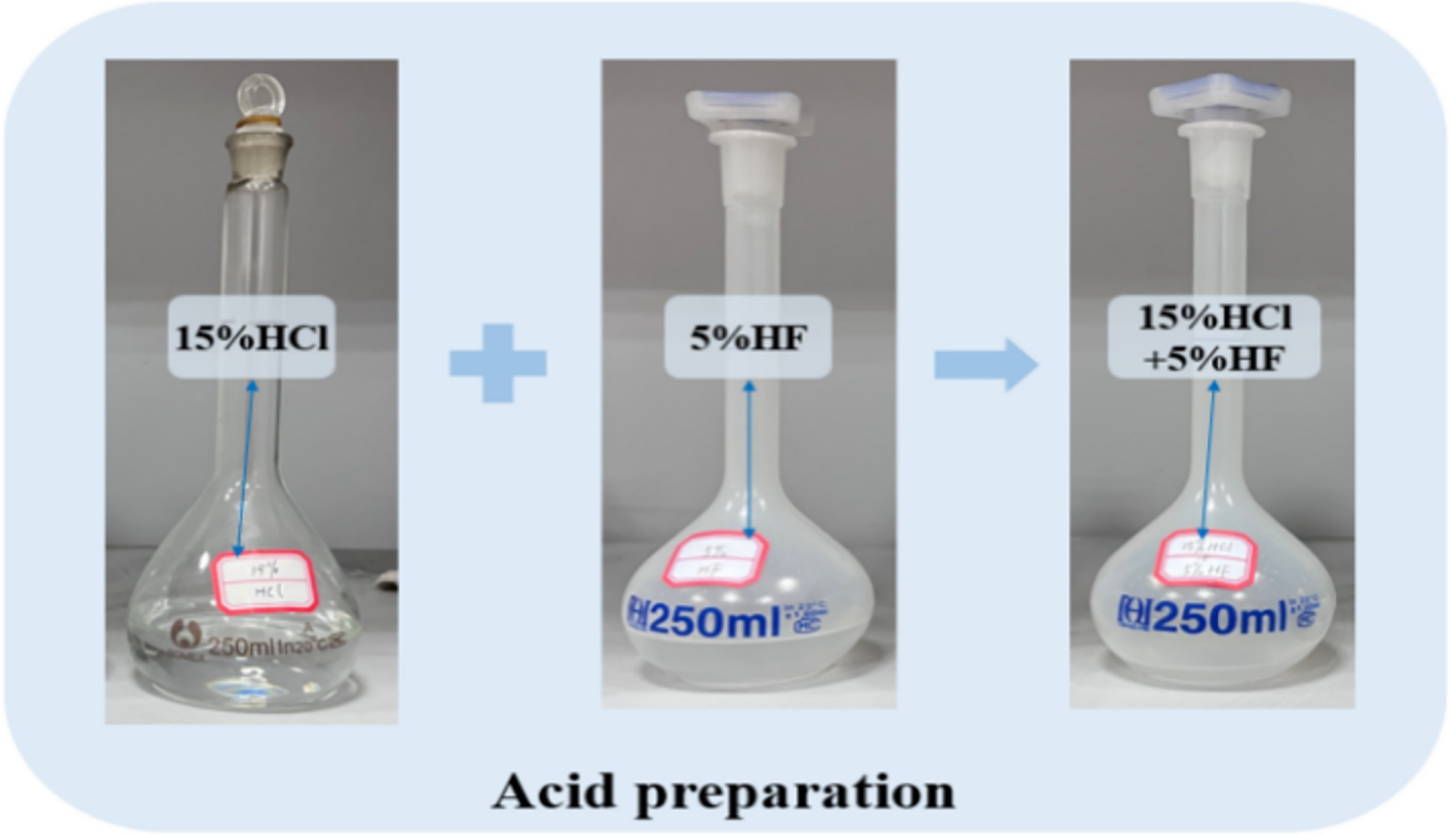
Acid preparation process.

Based on the XRD mapping, industrial composition, and elemental analysis results, Wenjiaba coal samples predominantly consist of quartz, kaolinite, and calcite, with carbon as the main element. A mixed acid solution comprising 15% HCL + 5% HF was chosen for the acidification process. This composition can effectively remove minerals from the pores and cracks of the coal [45, 46]. Fig 1 illustrates the prepared acid solution. Approximately 100 ml of acid solution was prepared, and the result was kept sealed.

#### 2.2.3 Acidification experiments

In acidification experiments, an extended reaction time can lead to a secondary reaction between the initial reaction product and the acid, resulting in the formation of a precipitate that may affect the experimental results [17]. Variations in temperature exert influence on the acidification effect [47, 48]. However, due to experimental constraints, the present study focuses solely on analyzing the acidification process at a temperature of 28°C. The dried particles of the coal samples and the standard samples were immersed in the prepared acid solution for 12 hours [49]. The coal samples were then filtered and dried using a dryer at a temperature of 60°C. Subsequently, the dried coal samples were stored in a sealed bag for preservation, ready for subsequent experiments.

#### 2.2.4 Experiments on the chemical structure of coal samples

The coal samples were initially crushed and sieved with a filter sieve to eliminate coal dust particles with a diameter greater than 0.0750mm. Subsequently, deashing was carried out, ensuring the preservation of the coal sample structure. The samples were then dried at 60°C in a constant-temperature drying oven for 2 hours until a constant weight was achieved. The XRD analysis was employed to examine the microcrystalline structure of the materials, the XRD parameters were as follows: tube pressure 40 KV, diffraction width DS = SS = 1°, scanning speed 2.000 (d/min), scanning range 10°~80°;a technique extensively used for the investigation of amorphous materials in recent years [50, 51]. Furthermore, FT-IR experiments were conducted to analyze the macroscopic chemical composition and microscopic molecular structure of the coal samples. The infrared spectroscopy experiment parameters were set as follows: resolution 4 cm^-1^, number of scans 16, scanning range 4000~400 cm^-1^.

#### 2.2.5 Experiments to analyze the coal sample pore structure

The capillary pressure curve obtained from the HP-MIP compression test provides fundamental parameters such as total porosity, pore size distribution, and specific surface area of the coal. The experiment was performed using an automatic mercury compression instrument (Mike AutoPore IV9500, Country), featuring a pressure range of 0.03–2200 MPa and a pore size range of 350~0.005 μm [52]. The mercury inlet pressure and pore radius r adhered to Washburn’s equation [53].


$$r=\frac{2\gamma \cos\theta }{p}$$

Where γ = 4.83×10^−3^ N/m is the surface tension of mercury; θ = 130° denotes the contact angle between mercury and coal sample surface; p represents the pressure of mercury.

The LT-N_2_A pore measurement range spans 0.9~400 nm, but it exhibits lower accuracy for large pores. In contrast, the pressed mercury method can offer more precise pore data. Consequently, low-temperature liquid nitrogen is employed to accurately measure the pore structure of coal samples. In this study, an automatic analyzer (Kantar AUTOSORB IQ, Country) was utilized to explore specific surface area and porosity. The coal samples were dried under vacuum conditions at 383 K for 8 hours to eliminate the influence of gas in the instrument on the results. Finally, the sorption and desorption curves of the coal samples were investigated at 77 K to obtain more accurate pore data.

#### 2.2.6 SEM experiments

The adsorption resolution capacity of coal is closely related to the pore structure, with the development degree and connectivity of pores in coal reservoirs directly influencing the adsorption, desorption, and diffusion of CBM. In this study, an SEM (ZEISS Sigma 300, Germany) was employed to analyze coal samples. The Wenjiaba tectonic coal before acidification and the Wenjiaba tectonic coal after acidification were magnified at 500 times, 2000 times, and 5000 times, respectively. This allowed for a more intuitive observation of the development of the pore and fracture structure of coal samples before and after acidification.

## 3 Results and discussions

In the experimental process, temperature emerges as a pivotal factor, exerting a direct influence on the kinetic process of acidification, encompassing both the reaction rate and the extent of acidification [47, 48]. Nevertheless, owing to constraints in our experimental setup, this study narrowly focuses on delineating the transformations in the physical pore structure and crystal structure of coal samples, both prior to and following acidification, within a 15% HCl + 5% HF acid solution maintained at a constant temperature of 28 degrees Celsius.

### 3.1 Characterization of the chemical structure of coal samples before and after acidification

In earlier studies, FTIR experiments were employed to investigate the alterations in functional groups before and after coal acidification, while XRD experiments were utilized to analyze the chemical composition of coal affected by acidification [54]. The content of functional groups vary with the acidification reaction time and the type of acid solution used [20, 55]. The macro chemical composition and micro molecular structure of coal were analyzed through XRD and FTIR experiments, yielding results consistent with those obtained in prior research.

#### 3.1.1 FT-IR experimental analysis

**Fig 2:**
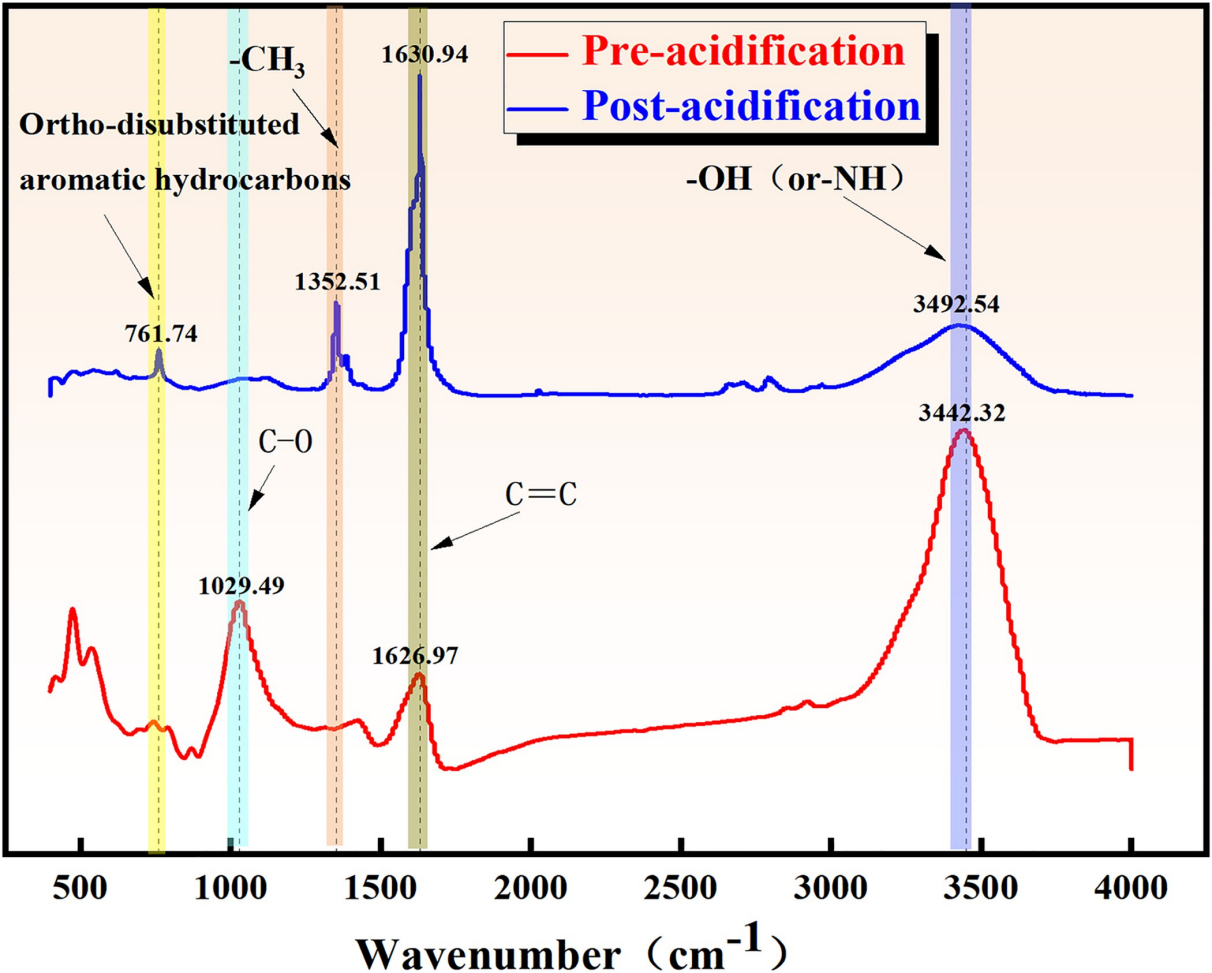
Infrared spectra of coal samples before and after acidification.

The chemical bonds and functional groups within the constituent coal samples vibrate constantly, with vibration frequencies comparable to the frequency of infrared light. When infrared light irradiates the molecules in the coal samples, different chemical bonds and functional groups absorb different frequencies, manifesting at distinct positions in the infrared spectral map. Fig 2 shows the Fourier infrared spectral map of Wenjiaba tectonic coal samples before and after acidification and transformation obtained through FT-IR experiments. The spectrograms were processed based on the absorption peaks attributed to the infrared spectra of coal, as illustrated in Table 3. The peak shapes were optimized, and the peaks, along with their corresponding functional groups were labeled.

**Table 3:** Absorption peaks in the infrared spectrum of coal.

Peaks	Absorption peak range(cm^-1^)	Spectral peak attribution
3400	3600~3200	Hydrogen bond-associated-OH (or-NH), phenol, alcohol, carboxylic acid
3030	3115~2990	Aromatic hydrocarbon CH vibration
2925	2943~2892	Asymmetric vibration of aliphatic CH_2_
2864	2875~2800	Asymmetric vibration of aliphatic CH_3_
2855	2875~2800	Symmetry vibration of aliphatic CH_2_
2660	2780~2350	Carbonyl (-C = O) Vibration
1900	2010~1880	Aromatic hydrocarbon, 1,2-disubstituted and 1,2,4-trisubstituted carbonyls
1700	1720~1687	Carbonyl (-C = O) vibration
1610	1645~1545	Aromatic C = C skeleton vibration
1460	1480~1421	Asymmetric vibration of CH_3_ and CH_2_ on the alkane chain structure
1375	1420~1350	symmetric bending vibration of—CH_3_
1318	1350~1210	Ar-O-C, R-O-C vibratory
1200	1200	(C_6_H_5_-OH)
1182	1210~1110	C-O of phenol, alcohol, ether, ester
1084	1110~1006	(-O-)
870	921~850	1,2,4-substituted aromatic hydrocarbons CH
810	850~800	Substitution of aromatic hydrocarbon CH
750	780~730	Ortho-disubstituted aromatic hydrocarbons

#### 3.1.2 Analysis of characteristic functional groups of coal

**Fig 3:**
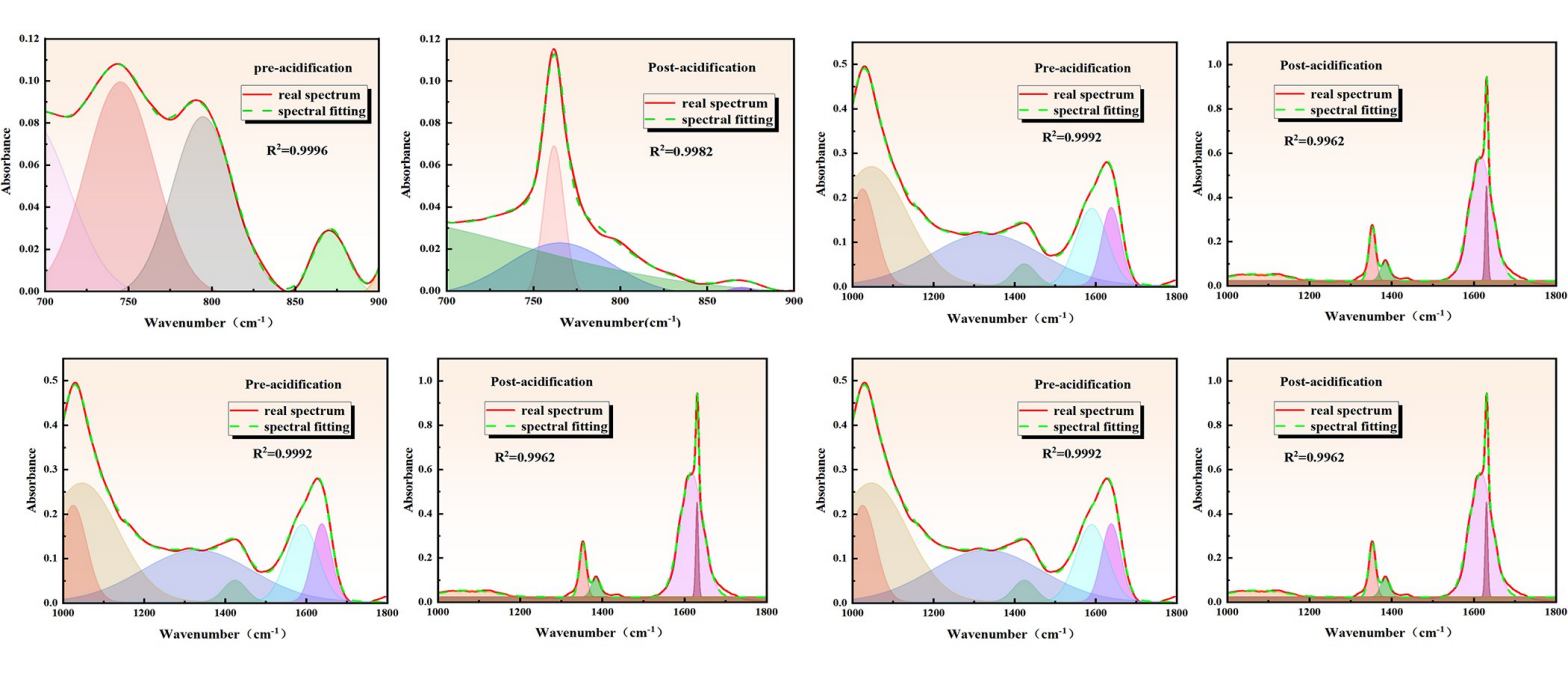
Infrared spectra peak fitting results before and after acidification. (a) Absorption peaks of aromatic structures before and after acidification. (b) Absorption peaks of oxygen-containing functional group structures before and after acidification. (c) Absorption peaks of aliphatic hydrocarbon structures before and after acidification. (d) Absorption peak fitting of hydroxyl structures before and after acidification.

According to the absorption peaks of infrared spectra, hydroxyl functional groups, aliphatic hydrocarbon structures, oxygenated functional groups, and aromatic structures were primarily distributed in the wavenumber range of 3000~3800 cm^-1^, 2800~3000 cm^-1^, 1000~1800 cm^-1^, and 700~900 cm^-1^, respectively. The infrared spectral curves for the aforementioned four regions were fitted with peaks, and the fitting results are illustrated in Fig 3.

1. *Aromatic structure analysis before and after acidification*. Acidification has the potential to enhance the coalification process, thereby affecting the molecular structures. The ensuing decarboxylation and condensation reactions increase the aromaticity of coal seams and the number of aromatic layers in the coal. The aromatic structure segments were identified through peak fitting of the infrared spectra of coal samples before and after acidification. Fig 3(A) reveals that the percentage of the absorption peak area at 630~692 cm^-1^ and 741~770 cm^-1^ increased from 14.75% and 44.07% to 48.45% and 50.04%, respectively, while the absorption peak area at 869–875 cm^-1^ decreased from 6.99% to 1.50%. This observation demonstrates that acidification enhances the aromatization degree.
2. *Structural analysis of oxygen-containing functional groups before and after acidification*. Oxygen-containing functional groups in coal are an accurate measure to determine the hydrophilicity and lipophilicity of coal. The primary regions of oxygen-containing functional groups in the infrared spectral map before and after acidification were fitted. Fig 3(B) shows that the percentage of absorption peak area at 1020~1072 cm^-1^ and 1320~1482 cm^-1^ decreased from 11.03% and 33% to 7.95% and 14%, respectively, while that at 1580~1640 cm^-1^ increased from 21.6% to 77%. Meanwhile, it is observed that the percentage of absorption peak area at 1640 cm^-1^ increased from 21.6% to 77.8%. The results demonstrate that the oxygen-containing functional groups in coal experienced varying degrees of reduction after acidification. However, the aromatic hydrocarbon C = C skeleton vibration and the structure of CH_3_ and CH_2_ asymmetric vibration on the alkane chain structure were strengthened.
3. *Structural analysis of aliphatic hydrocarbons before and after acidification*. Before acidification, the 2800~3000 region is primarily associated with aliphatic CH_2_ symmetric and asymmetric vibration, as well as aliphatic CH_3_ asymmetric vibration. Fig 3(C) shows that the percentage of absorption peak area at 2929~2970 cm^-1^ decreases from 93.46% to 22. 49%, and the wave peak area at 2863~2889 cm^-1^ disappears after acidification. This observation indicates that the acidification process weakens the aliphatic CH_2_ asymmetric vibration structure, as well as the aliphatic hydrocarbon CH_3_ asymmetric vibration and CH_2_ symmetric vibration.
4. *Structural analysis of hydroxyl groups and hydrogen bonding groups before and after acidification*. The hydroxyl functional group in coal is hydrophilic and acidophilic, capable of forming hydrogen bonds and van der Waals forces with methane molecules. The content of hydroxyl groups significantly influences methane adsorption and desorption. Fig 3(D) indicates that the wave area of the fitted curve after acidification modification increased from 305.35 cm^2^ to 74 cm^2^ in the band 3200~3600 cm^-1^. This observation demonstrates that the acidification process weakened the structure of hydroxyl and other hydroxyl groups involved in hydrogen bonding in the experimental coal samples.

In summary, the acidification and modification experiments significantly affected the internal structure of Wenjiaba tectonic coal. This transformation primarily involves the reduction of structures such as hydroxyl groups and oxygenated functional groups that participate in hydrogen bonding within the coal, along with an enhancement in the degree of aromatization.

#### 3.1.3 Analysis of chemical structure parameters

Chemical structures in coal before and after acidification can be quantified using aromaticity *f*_*a*_ and fat-branched chain length *R*. These parameters are defined as follows:

$$\frac{H_{al}}{H}=\frac{H_{al}}{H_{al}+H_{ar}}=\frac{A_{2800∼3000}}{A_{2800∼3000}+A_{100∼900}}$$

$$f_{a}=1-(\frac{H_{al}}{H}\times \frac{H}{C})/\frac{H_{al}}{C_{al}}$$

$$R=\frac{A({\mathrm{CH}}_{2})}{A({\mathrm{CH}}_{3})}=\frac{A_{2935∼2990}}{A_{2800∼2920}}$$

where *A*_700~900_, *A*_2800~3000_, *A*_2935~2990_, and *A*_2800~2920_ correspond to the areas occupied by spectrally absorbed light in the wavenumber bands 700~900 cm^-1^, 2800~3000 cm^-1^, 2935~2990 cm^-1,^ and 2800~2920 cm^-1^, respectively. The ratio of hydrogen to carbon (*H/C*) can be obtained from the results of elemental analysis. The ratio of hydrogen in aliphatic structures to carbon (*H*_*al*_*/C*_*al*_) is assumed to be 1.8 [56]. Moreover, the relative abundance of hydrogen (*I*) and the structural parameter (C) for both aromatic and aliphatic hydrocarbons can be computed using Eqs (5) and (6), respectively [57, 58].

$$I=\frac{A_{700∼900}}{A_{2800∼3000}}$$

$$‘C’=\frac{A_{1000∼1400}{+A}_{1630∼1730}}{A_{1000∼1400}{+A}_{1630∼1730}{+A}_{1600}}$$

where *A*_1000~1400_, *A*_1630~1730_, and *A*_1600_ correspond to the integral area of the spectral absorption band at wavenumber of 1000~1400 cm^-1^, 1630~1730 cm^-1^, and 1600 cm^-1^, respectively. Table 4 provides the chemical elemental parameters of coal before and after acidification obtained using Eqs (2)-(6).

**Table 4:** Chemical structure parameters of coal sample before and after acidification.

Coal samples	*f*_*a*_	*R*	*I*	‘*C*’
Pre-acidification	0.889	2.708	0.845	0.862
Post-acidification	0.967	1.112	1.275	0.934

Table 4 reveals that the reaction between the experimentally proportioned acid and the chemical constituents in the coal samples affects the parameters of aromaticity (f_a_) and aliphatic branched-chain length (*R*), as well as the relative abundance of hydrogen in aromatic and aliphatic hydrocarbons (I), and the structural parameter of oxygen-containing functional groups (*C*). In comparison with the pre-acidification period, *f*_*a*_ increased by 8.77%, *R* decreased by 58.94%, *I* increased by 50.80%, and *C* increased by 7.48%. Notably, the parameter *R* exhibited the most significant change, demonstrating that acidification weakened the aliphatic CH_2_ asymmetric vibrational structure, as well as the aliphatic hydrocarbon CH_3_ asymmetric vibration and CH_2_ symmetric vibration in the coal samples.

#### 3.1.4 X-ray diffraction experiments

**Fig 4:**
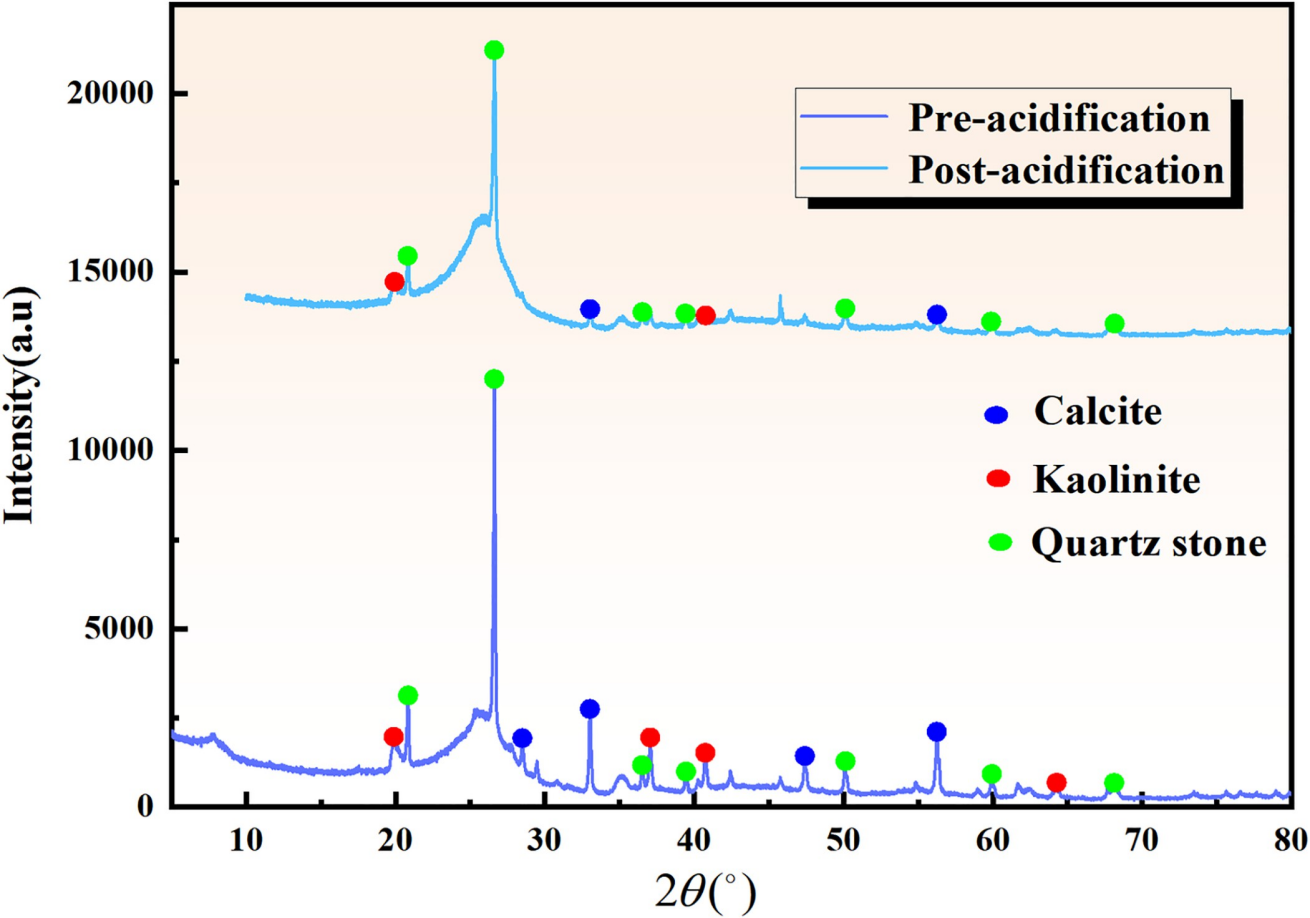
Coal mineral composition before and after acidification.

XRD experiments were performed on coal samples, and the XRD spectra of coal samples before and after acidification were analyzed using MDI (Jade 6.0) software based on the position and intensity of standardized peaks with corresponding components. Fig 4 indicates that the primary mineral components identified in coal samples included quartz, kaolinite, and calcite.

**Fig 5:**
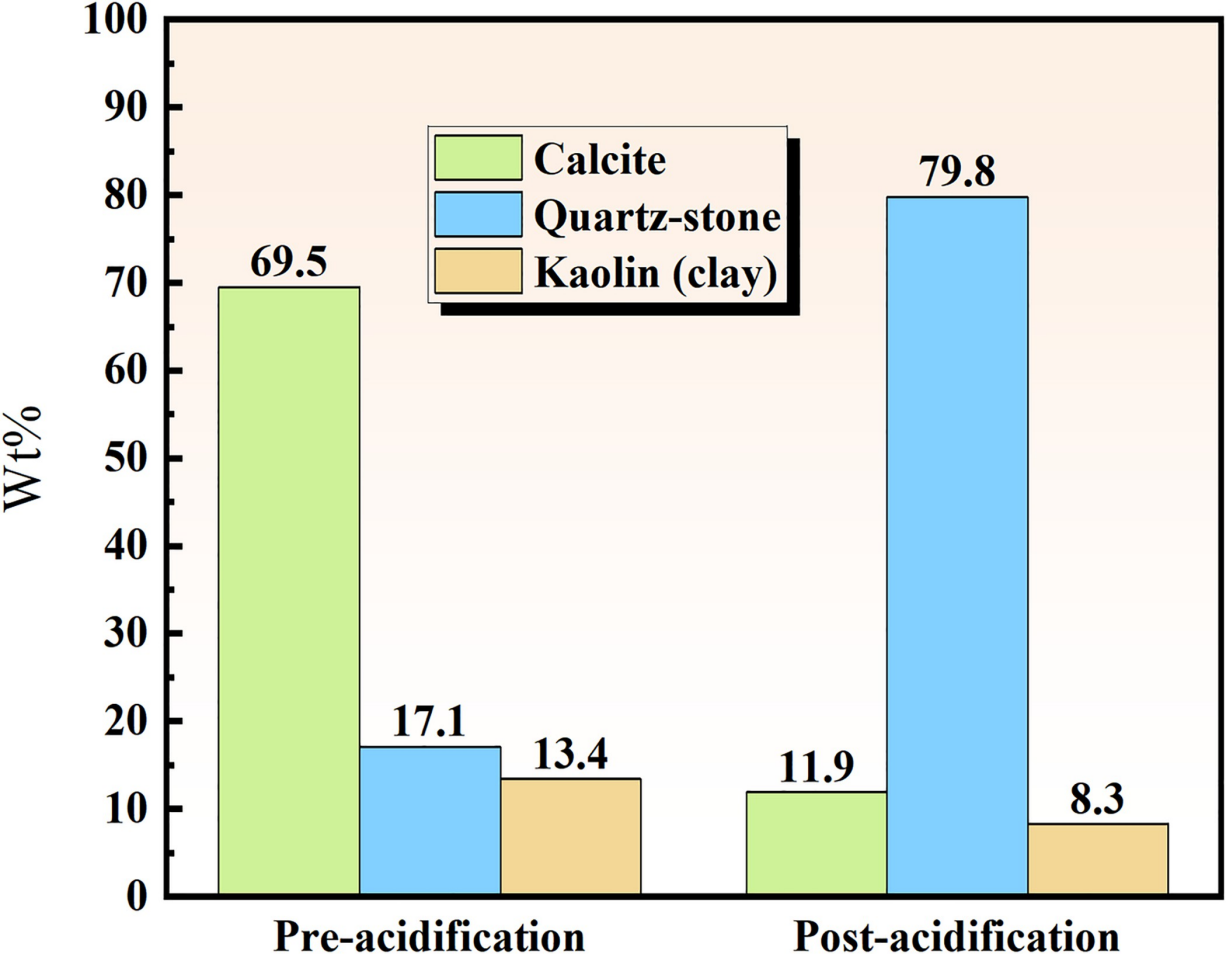
The mineral content of coal before and after acidification.

Due to the chemical reaction between the acid solution and the minerals in the coal sample, there was a noticeable decrease in the diffraction intensity of kaolinite and quartz in the coal sample after acidification compared to before acidification. Particularly, Fig 5 shows that some diffraction peaks disappeared. The acidification process reduced calcite and kaolinite content in coal samples, while the stability of quartzite remained strong, making it resistant to complete dissolution by the acid. Consequently, a significant portion of quartzite remained in the coal samples after acidification. It is inferred that some kaolinite, quartz, and calcite were dissolved by the acid.

#### 3.1.5 Crystal structure analysis

To understand the structural parameters of the coal samples before and after acidification, the XRD patterns were analyzed to examine the crystalline structure of the coal. The diffraction peaks of the coal’s crystalline structure were identified at the same positions as the graphite (002) and (100) crystal planes when subjected to XRD. The impact of acidification on the crystalline structure of coal can be evaluated through the comparison of the results [59, 60]. Parameters such as inter-crystalline spacing (d_002_), diameter, height of the microcrystals, and Lc were calculated using the Bragg-Scherrer equation [55].

$$d_{002}=\frac{\lambda }{2\sin\theta_{002}}$$

$$L_{c}=\frac{0.9\lambda }{\beta_{002}\cos\theta_{002}}$$

$$L_{a}=\frac{1.84\lambda }{\beta_{100}\cos_{100}}$$

$$f_{a‐\mathrm{XRD}}=\frac{A_{002}}{A_{002}+A_{\gamma }}$$

where *λ* is the wavenumber of X-rays, taken as 0.15405 nm; *θ*_002_ and *θ*_100_ are the angles corresponding to the positions of peaks 002 and 100, respectively. *β*_002_ and *β*_100_ denote the half-peak full width of peaks 002 and 100, respectively; *A*_002_ and *A*_*γ*_ represent the area of peaks 002 and 100, respectively; *f*_*a*−*XRD*_ is the aromaticity.

**Fig 6:**
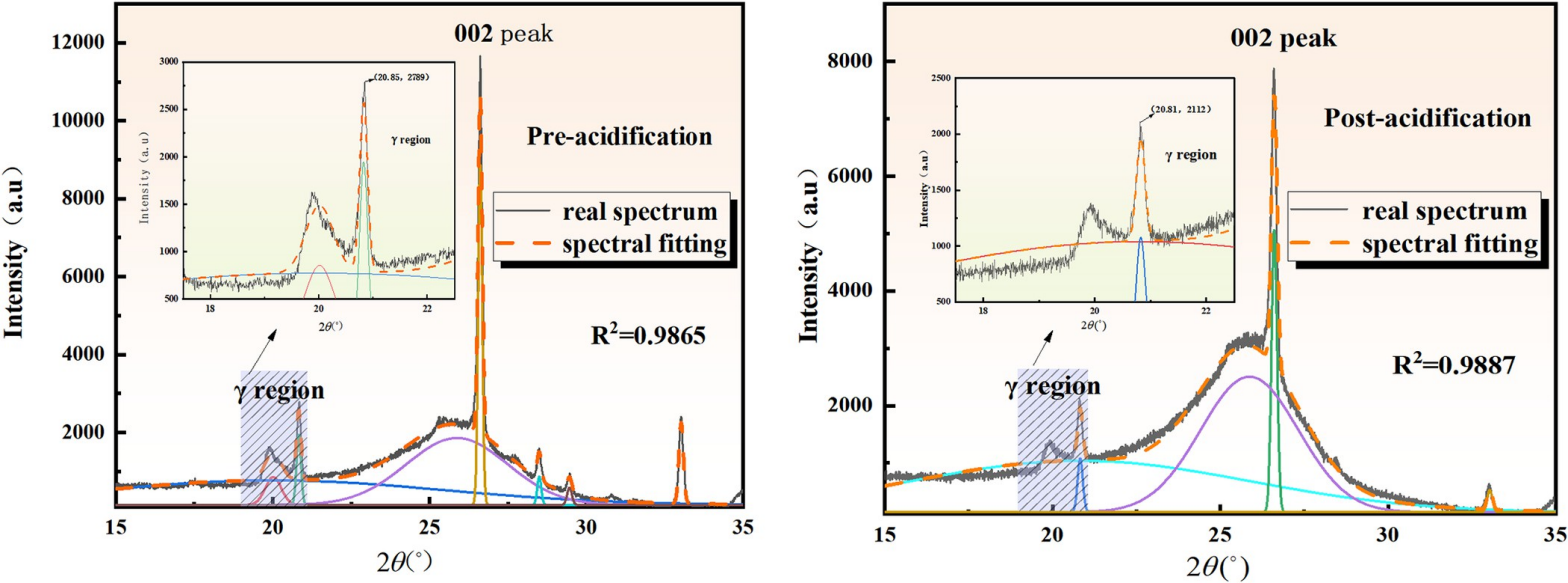
Fitting of peak 002-XRD spectra before and after acidification.

Fig 6 illustrates the peak-fitted XRD spectra of the 002 peak of the coal samples before and after acidification.

Fig 6 reveals that the γ peak, corresponding to the diffraction peak in the fat group of coal, exhibits a positive correlation with its structure [61]. It is observed that the diffraction intensity of the γ peak reduces by 24.27% after acidification, indicating a corresponding decrease in the fat structure intensity of the samples. The data obtained through Gaussian peak fitting were incorporated into Eqs (7)- (10) to derive the inter-crystal spacing d_002_, the average diameter and height of the microcrystals, and the aromaticity. The results are presented in Table 5.

**Table 5:** Crystal structure parameters of coal samples before and after acidification.

Coal samples	2*θ*(°)	*d*_*002*_	*L*_*a*_	*L*_*c*_	*f*_*a-XRD*_
Pre-acidification	26.616	0.3346	1.959	0.792	0.943
Post-acidification	26.609	0.3347	1.641	0.759	0.979

Table 5 indicates that acidification damaged the crystal structure of the coal samples, affecting inter-crystal spacing, diameter, height, and aromaticity of the coal samples before and after acidification. The results demonstrate that *d*_*002*_ did not change significantly and fa-XRD increased by 3.7%, while *L*_*a*_ and *L*_*c*_ decreased by 16.23% and 4.17%, respectively. The substantial decrease in La suggests that the acid proportion damaged the crystals of the coal samples, primarily in terms of lateral corrosion. Moreover, the increase in aromaticity after acidification indicates that acidification enhances the aromatic structure of the coal samples, which is consistent with the observations from the infrared spectroscopy experiments.

### 3.2 Pore structure characteristics of coal before and after acidification

The porosity and fracture structure of coal significantly impacts the content of free gas in coal reservoirs. In this context, HP-MIP and low-temperature liquid nitrogen experiments are typically employed to investigate the pore and fracture structure of materials. The mercury injection curve can directly indicate the development and connectivity of pores and fractures in coal samples [62]. Accordingly, the present study conducted HP-MIP and low-temperature liquid nitrogen experiments to explore the pore structure of coal before and after acidification. The results were compared with experimental data [63–65].

#### 3.2.1 High-pressure mercury compression experiments

The coal samples before and after acidification were utilized in high-pressure mercury pressure experiments. The capillary pressure curves measured during these experiments provided essential parameters such as total porosity, pore size distribution, and specific surface area of the coal, as presented in Table 6. It is observed that after acidification, the Wenjiaba tectonic coal exhibited significantly larger porosity and mercury input compared to those before acidification. Moreover, the average pore size of the coal samples before acidification was 205.1 nm, while it increased by 244.11% during acidification. However, the specific surface area was smaller than that of the coal samples before acidification. This relationship between porosity, specific surface area, and total area during the acidification process indicates a negative correlation between porosity and specific surface area. Consequently, when porosity increases and the total area of the coal sample remains constant after acidification, there is a negative correlation between the porosity and the specific surface area. The changes in pore structure after the acidification transformation experiment indicate enhanced pore development, with a noticeable trend of increased average pore diameter, demonstrating an increase in micropores and transition pores due to the transformation effect.

**Table 6:** Parameters of the pore structure within coal samples.

Coal samples	Porosity (%)	Mercury intake×10^−2^ (mL/g)	Specific surface area (m^2^/g)	Average pore size (nm)
Post-acidification	44.9976	0.5197	4.152	500.66
Pre-acidification	32.0342	0.2370	4.623	205.1

**Fig 7:**
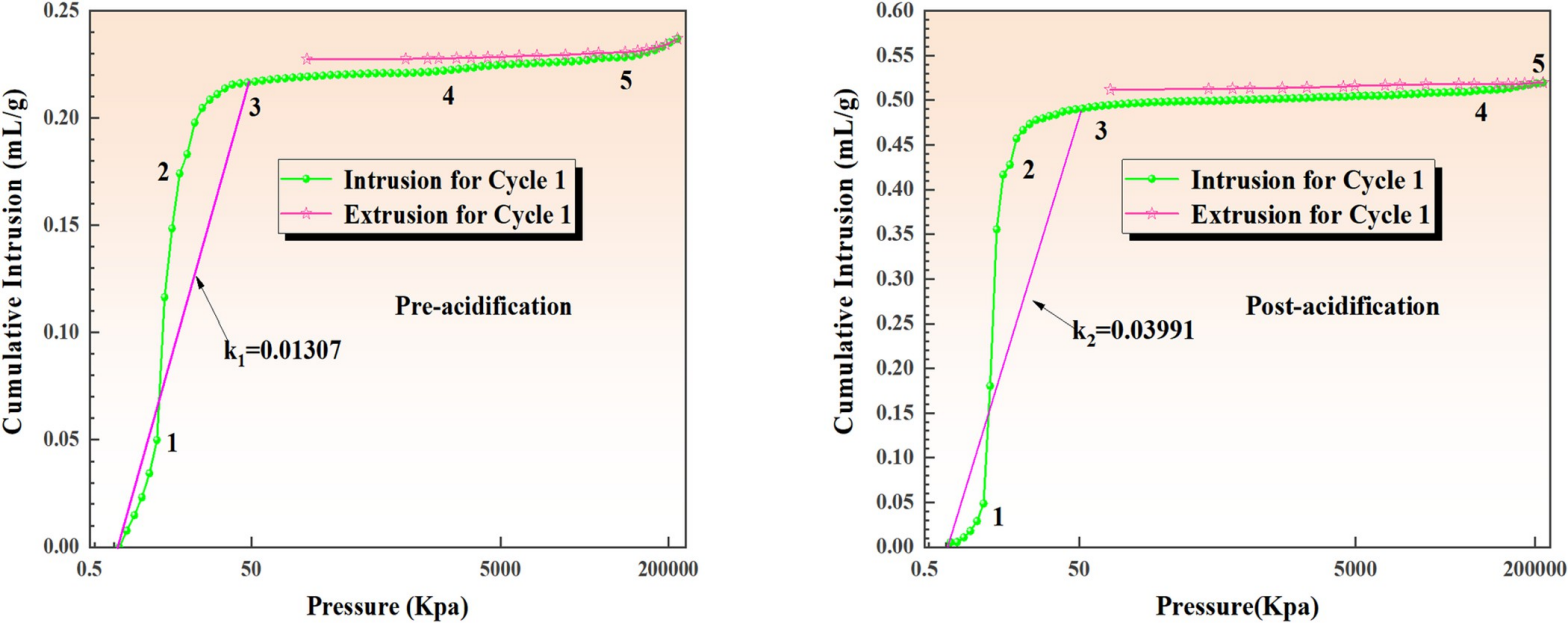
Mercury advance and retreat curves of tectonic coal before and after acidification.

*3*.*2*.*1*.*1 Analysis of mercury supply curves*. The mercury supply curve effectively and intuitively reflects the internal pore development and connectivity of coal samples. Fig 7 illustrates the mercury supply curves for coal samples before and after acidification.

Analyze the results are as follows:

1. As the pressure increases, the cumulative amount of mercury supply gradually rises and eventually reaches a smooth curve. The more complete curve represents the mercury feed curve under the condition of pressure increase, while the other curve is the mercury discharge curve.
2. Fig 7 shows five stages of the mercury curve, divided by the inflection point position. Stages 1–3 primarily involve the pore capacity between coal particles, showing a gradual increase in the amount of mercury with the slow rise in pressure. In stages 3 and 4, the amount of mercury increases without a significant rise in energy consumption as the coal particles are compressed. Stages 4 and 5 involve a larger increase in pressure, where particles absorb more energy, leading to further compression of volume.
3. Additionally, high-pressure mercury was injected into capillary and microscopic pores of coal samples. Beyond the fifth stage, even smaller pores were injected with high-pressure mercury, resulting in a cumulative mercury feed amount of about 0.23 mL/g. In the acidified coal samples, the mercury injection amount under the same pressure conditions was approximately 0.23 mL/g. However, after acidification and transformation, the cumulative mercury feed amount of coal samples significantly increased and reached 0.5 mL/g under the same pressure conditions. This was approximately twice the cumulative mercury feed amount observed before acidification and transformation. Moreover, the analysis of the steepness of the curve revealed that the mercury feed curve after acidification was steeper than the slope before acidification.
4. Analyzing the slope curves of stages 1–3 reveals that the slope values before and after acidification are k1 = 0.01307 and k_2_ = 0.03991, respectively. This indicates that acidification modification enhances the degree of pore development, leading to structural changes in the pores of coal samples. As a result, the amount of mercury absorbed in coal samples increases, and more micropores and transition pores are released, thereby improving the desorption capacity of the samples.

**Fig 8:**
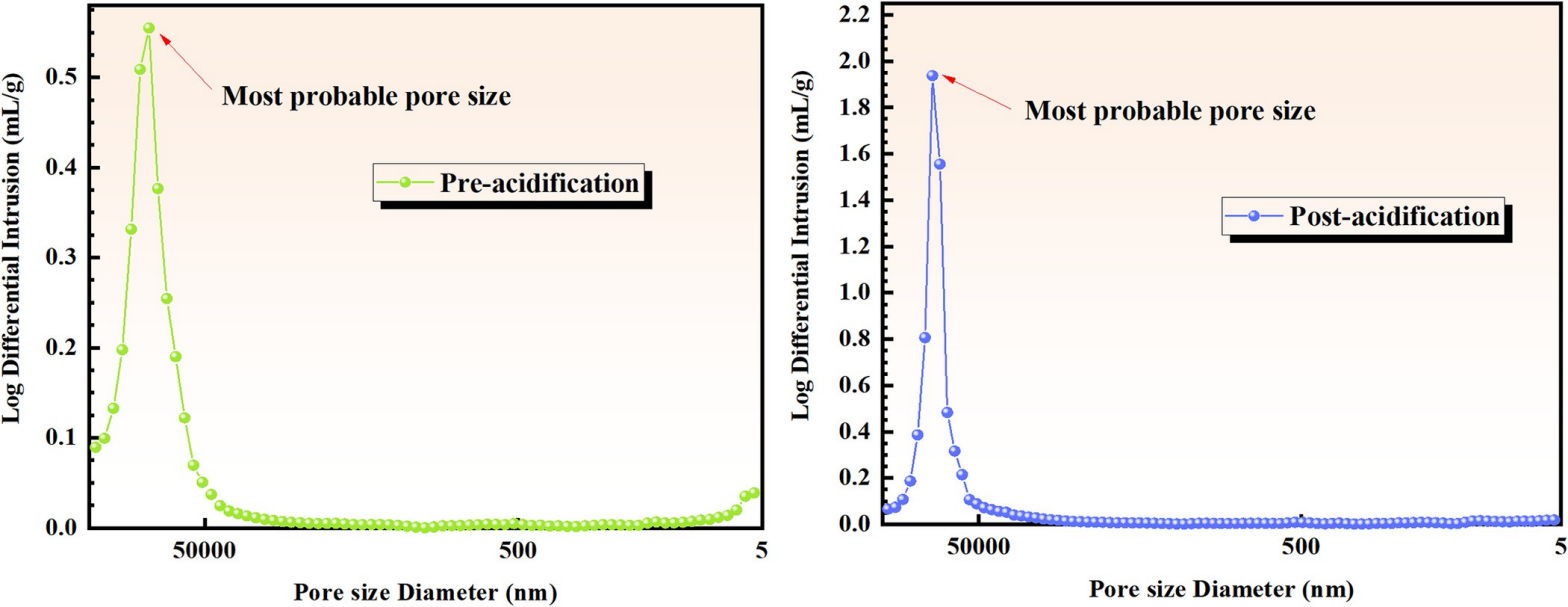
Distribution of logarithmic differential volume versus pore size before and after acidification.

Fig 8 illustrates the relationship between logarithmic differential volume and pore size of coal samples before and after acidification.

Fig 8 shows that the most available pore size of the coal sample before and after acidification is 0.55 and 1.85, respectively. This observation represents an over threefold increase compared to the pre-acidification state. The enhanced range of the most distributed pores in the coal samples after acidification indicates a significant improvement, and the desorption capacity of the coal samples primarily occurs through micropores and transition pores. It is worth noting that the number of micropores and transition pores is of significant importance in determining the desorption capacity of the coal. The results demonstrate that the acidification modification experiment increases the number of micropores and transition pores, thereby promoting the desorption capacity of coal samples.

#### 3.2.2 Microporous structure of coal

**Fig 9:**
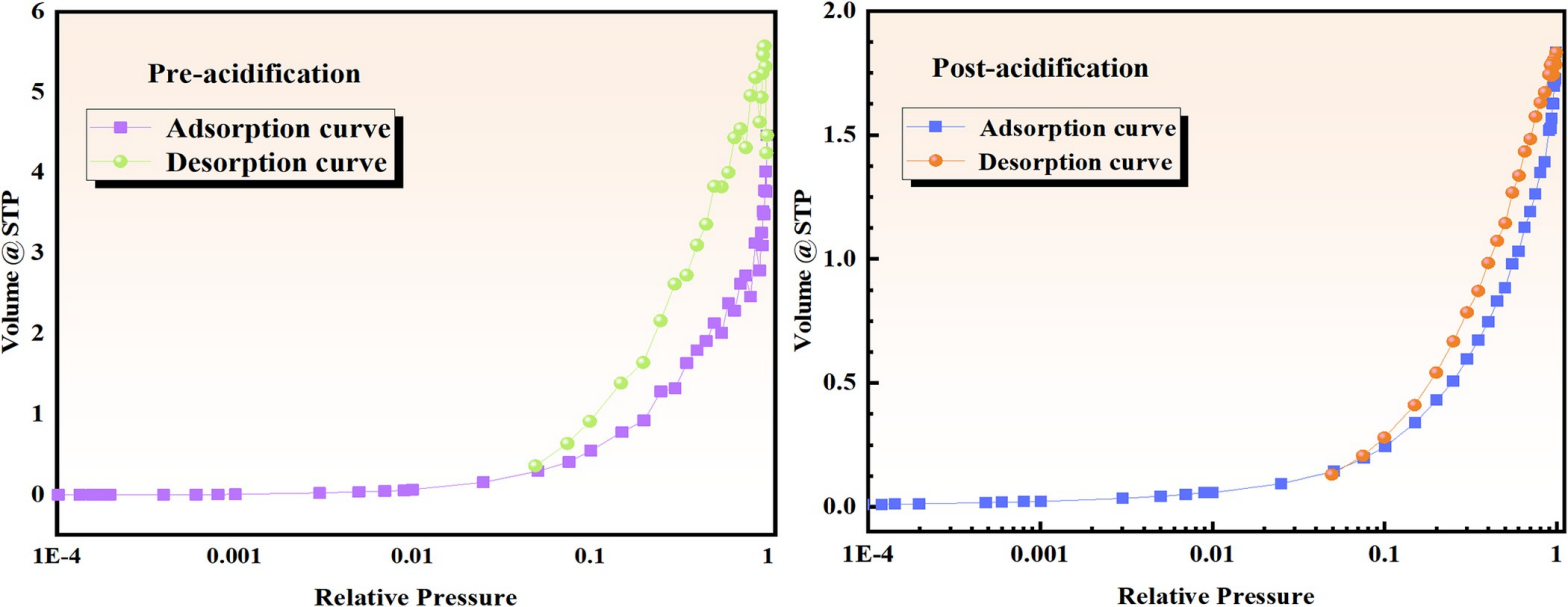
Absorption and desorption curves of coal samples before and after acidification.

In this section, the experimental data of Feng Cong et al. [66]. are used for verification. The correlation between adsorbate and pressure in a specific system under constant temperature and relative equilibrium is referred to as the adsorption isotherm. The results are presented in Fig 9.

Before acidification, the coal samples exhibited an adsorption and desorption capacity of approximately 5.5, whereas this capacity was reduced to 1.8 after acidification. A larger adsorption capacity indicates greater pore space and stronger adsorption capabilities in coal samples. Although the adsorption capacity decreased notably after acidification, it still maintained a class IV(a) adsorption type. This suggests that acidification modification enhanced the desorption capacity of the coal samples.

The adsorption isotherm analysis revealed a hysteresis loop within the range of 0.5<P/P_0_<0.99, indicating that the evaporation in the pore differs from the condensation within it, reflecting the capillary condensation phenomenon in the presence of mesopores [67]. The hysteresis type of coal samples, classified according to the ISO 9277:2022 recommendations by IUPAC [68], was identified as H3 type. This classification indicates the presence of a significant number of open pores in the samples, providing more intuitive insight into the adsorption isotherms of coal samples.

*3*.*2*.*2*.*1 Characteristics of adsorbed pore surface area and pore volume parameters of coal samples before and after acidification*. According to the BJH model, the surface area of various types of pores in the coal samples, before and after acidification, can be categorized and summarized in Table 7. Moreover, the pore volume data before and after acidification are presented in Table 8. It is observed that the surface area of micropores and transition pores in the coal samples before acidification constituted over 93% of the total pore surface area. Following acidification, there was a significant reduction in the surface area of micropores and transition pores, but their percentage contribution did not change markedly. The maximum cumulative pore volume of the coal sample after acidification was approximately 0.0025, representing a reduction of about 68.75% compared to that before acidification. The pore size distribution curves of micropores and transition pores also demonstrated a reduction of about 70% after acidification, indicating that acidification modification, along with the coal composition, played a significant role in increasing pore volume, thereby reducing the proportion of micropores.

**Table 7:** Pore area of coal samples before and after acidification.

Coal samples	Surface area of pores (m^2^·g^-1^)				Percentage of surface area of each pore(%)		
	Microvias	halfway hole	Medium holes	Total holes	Microvias	Transition holes	Medium holes
Pre-acidification	3.604	6.003	0.677	10.284	35.04	58.37	6.59
Post-acidification	1.032	1.792	0.209	3.033	34.03	59.08	6.89

**Table 8:** Pore volume of coal samples before and after acidification.

Coal samples	Pore volume(cm^3^·g^-1^)				Percentage of volume of each hole(%)		
	Microvias	halfway hole	Medium holes	Total holes	Microvias	halfway hole	Medium holes
Pre-acidification	0.03707	0.07588	0.01040	0.12335	30.05	61.52	8.43
Post-acidification	0.01034	0.02958	0.00459	0.04451	23.23	66.46	10.31

**Fig 10:**
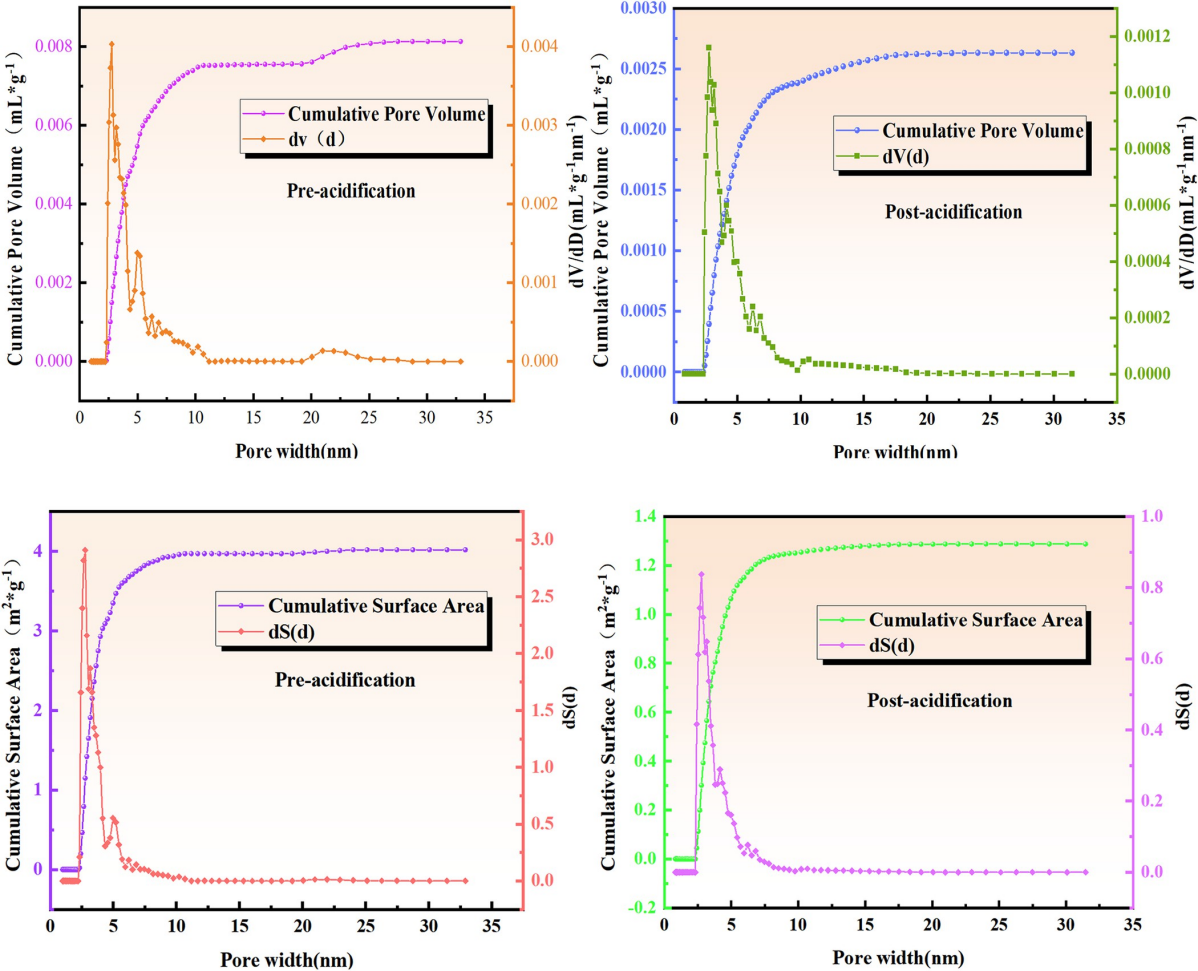
Pore structure and pore size distribution of coal samples before and after acidification. (a) Pore size distribution of coal sample before and after acidification. (b) Pore structure parameters of coal sample before and after acidification.

Fig 10 shows the pore structure parameters and pore size distribution of the coal samples before and after acidification. It is noteworthy that the analysis in this section focuses solely on micropores and transition pores, as cryogenic liquid nitrogen is primarily employed to analyze these specific pore types.

Fig 10(A) indicates that the cumulative pore volume after acidification approaches its value of 0.0025, representing a reduction of 68.75% compared to the pre-acidification period. The pore size distribution curves for micropores and transition pores also indicate a decrease of about 70% in pore size distribution after acidification compared to the pre-acidification period. This observation demonstrates that acidification modification and the material composition of the coal samples affect chemical interactions within coal, resulting in increased pore volume and a subsequent decrease in the proportion of micropores. Fig 10(B) shows the pore structure parameters of the coal samples before and after acidification. It is found that the cumulative specific surface area after acidification is reduced by approximately 67.5% compared to the pre-acidification period. This supports the conclusion that acidification modification engages in a chemical interaction with the material composition of the experimental coal samples, leading to increased pore volume and a reduction in the proportion of micropores.

#### 3.2.3 SEM experimental analysis

**Fig 11:**
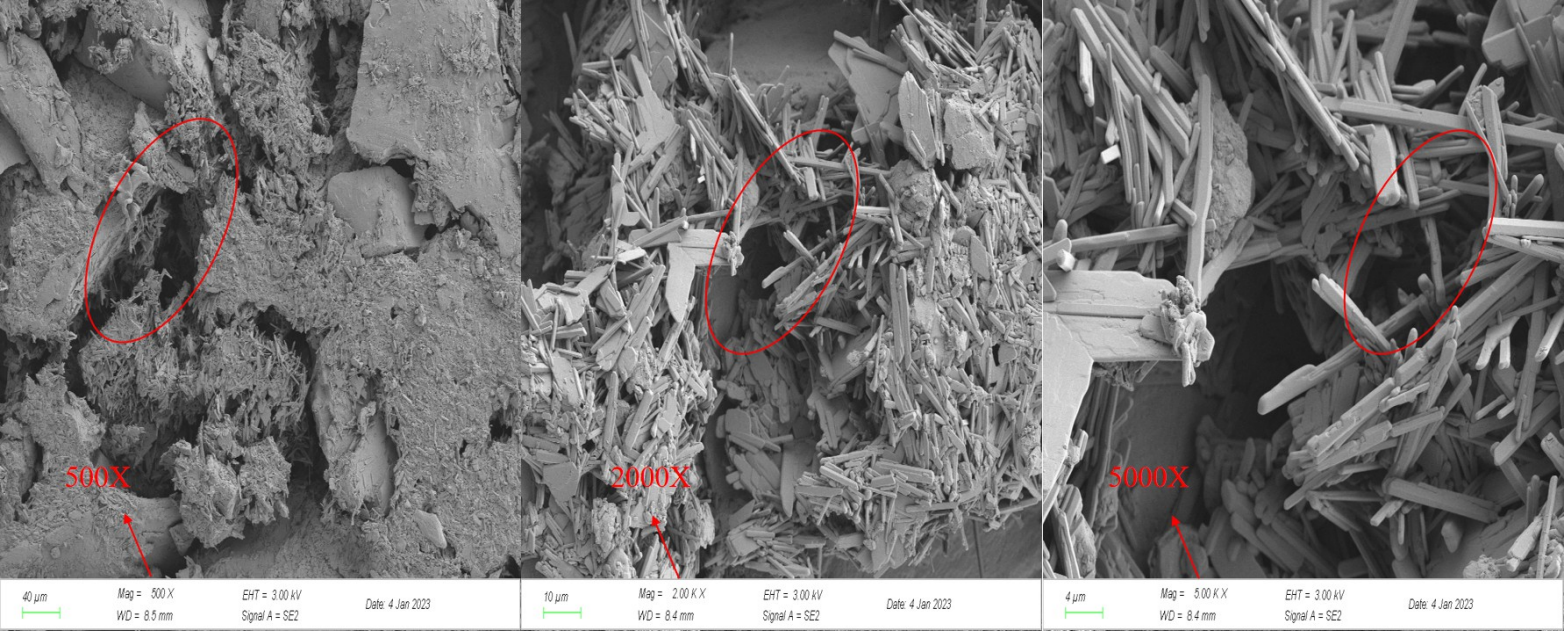
SEM image of coal sample before acidification.

**Fig 12:**
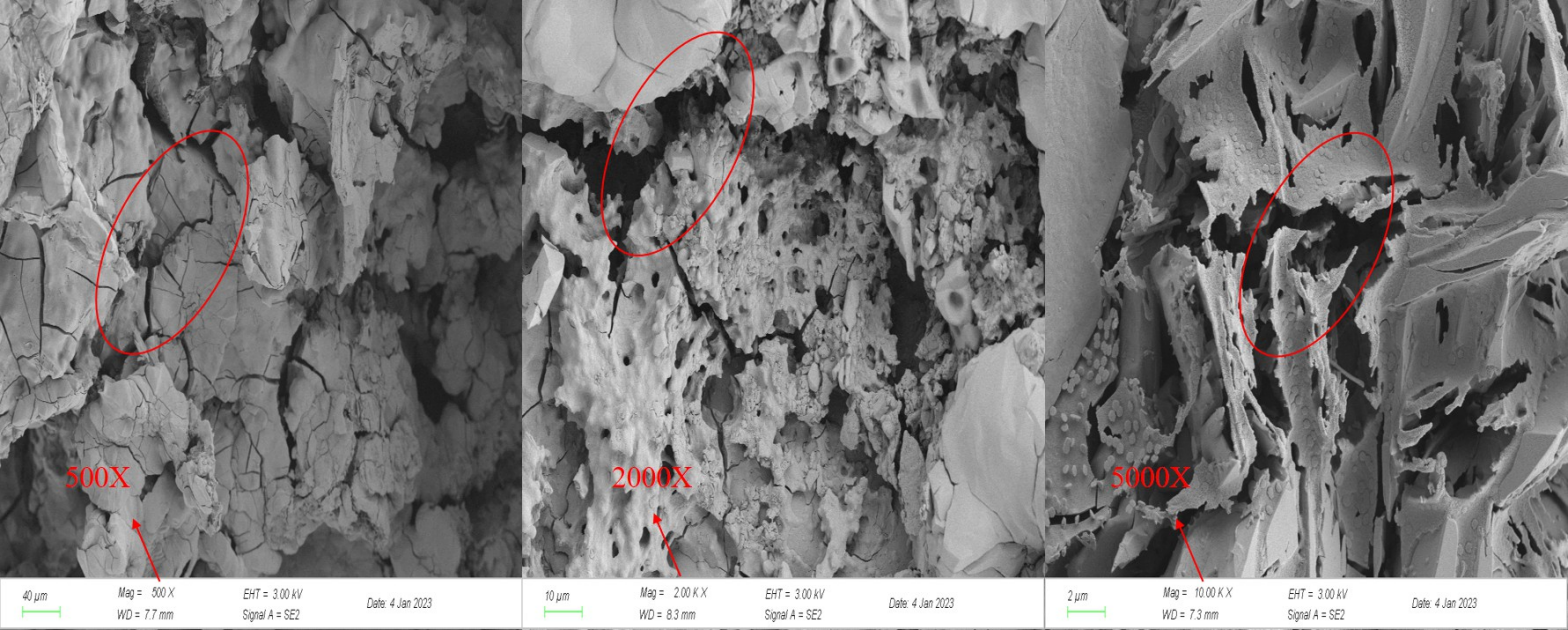
SEM image of coal samples after acidification.

To study the effect of acidification on the evolution of pore and fissure structure of coal samples, the samples before and after acidification were magnified 500 times, 2000 times, and 5000 times, as shown in Figs 11 and 12.

Fig 11 presents the SEM images before acidification, revealing that in the 500X magnified image, the surface of the coal sample appears rough, and the pores and fissures are not prominently visible. In the 2000X and 5000X magnified images, the minerals filling the pores and fissures of the coal sample are evident. Conversely, the SEM images in Fig 12 demonstrate a smooth surface of the coal sample after acidification with distinct visibility of the pore and fissure structure. In the 2000X and 5000X magnified images, the cracks and pores on the surface of the coal samples are prominently observed. These observations suggest that the acid treatment corroded the mineral components that filled the pores and cracks within coal samples, leading to an increase in pores and cracks. The presence of these pores increases the specific surface area of the coal samples, aligning with the outcomes of Hg-pressure and BET experiments.

## 4 Conclusions

The current study endeavors to delve into the intricate pore and fracture structure, along with the molecular composition of coal samples, prior to and following the process of acidification. While variations in temperature can potentially alter the outcomes of acidification experiments, our exploration has been circumscribed to the specific outcomes achieved at a constant temperature of 28°C, owing to the constraints of our experimental setup. Drawing upon the insights garnered from our findings and comprehensive analysis, the pivotal advancements of this investigation can be distilled as follows:

1. The acid-induced alteration in the coal samples involves the destruction of the crystal structure, primarily characterized by lateral corrosion. This process enhances the aromaticity of the coal samples and increases the relative abundance of hydrogen in aromatic and aliphatic hydrocarbons. Simultaneously, it weakens the structure related to aliphatic CH_2_ asymmetric vibration, as well as aliphatic hydrocarbon CH_3_ asymmetric vibration and CH_2_ symmetric vibration in the coal samples. Additionally, the structure of hydroxyl and oxygen-containing functional groups is reduced. This reduction is particularly more pronounced in structures associated with hydrogen bonding.
2. After acidification, the porosity and mercury uptake of the coal samples significantly increased compared to those before acidification. The average pore size of the coal samples after acidification was 500.66 nm, showing a 244.11% increase over the average pore size before acidification. Moreover, the most available pore size of the coal samples after acidification exhibited a 300% increase relative to the pre-acidification period, indicating a substantial enhancement in the range of pore sizes with the highest distribution in the coal samples after acidification.
3. The maximum cumulative pore volume after acidification is approximately 0.0025, representing a reduction of about 68.75% compared to the pre-acidification period. The pore size distribution curves of micropores and excess pores also indicate a decrease of about 70% in the distribution of pore sizes after acidification compared to that before acidification. This observation demonstrates that the acidification process affects the material composition of coal, increasing pore volume and consequently reducing the proportion of micropores. The cumulative specific surface area after acidification decreased by about 67.5% compared to before acidification. This finding demonstrates that acidification modification interacts chemically with the material composition of the coal samples, leading to increased pore volume and a lower percentage of micropores.
4. The SEM results provide a more intuitive demonstration that acidification enhanced the pore and fracture structure of the coal samples. It corroded the minerals filling the pores and fractures, resulting in increased fracture width and texture. This observation illustrates that acidification improved the connectivity of the pore and fracture structure in the coal samples. This improvement is crucial for enhancing the production capacity of CBM in subsequent processes.
5. Acidification not only affects the content of chemical functional groups in coal but also corrodes minerals present in the pore and fissure structure, enhancing the connectivity of the pore and fissure network in coal. This provides a reliable theoretical approach for pumping and increasing the production of CBM.

In future research, the exploration of different acid solutions or the examination of the influence of strong alkaline solutions on coal samples will be conducted. Additionally, the investigation will focus on assessing whether the efficiency of the acidification process can be improved through the use of catalysts. These endeavors are expected to provide theoretical guidance and experimental insights for the future development of CBM in Guizhou.

Furthermore, to address the adverse effects of mixed acid on coal quality and the environment, the following four measures can be implemented. a)Implementation of coal mine emission control: This involves the efficient collection and treatment of acidic wastewater and waste gas produced during coal mine production processes to mitigate and minimize acid emissions. b)Waste Management: Ensuring proper treatment and disposal of waste generated during coal mine production to prevent soil and water pollution caused by acidic waste. c)Environmental Monitoring: Implementing a comprehensive environmental monitoring system to regularly assess the quality of soil, water, and air in the vicinity of the coal mine. This ensures timely identification and resolution of issues related to the environmental impact of acid. d)Technological Improvement: Implementing advancements and innovations in technology to minimize acid generation during coal mine production. This includes enhancing resource utilization efficiency and mitigating environmental impact.

## Supporting information

S1 Data (ZIP)
